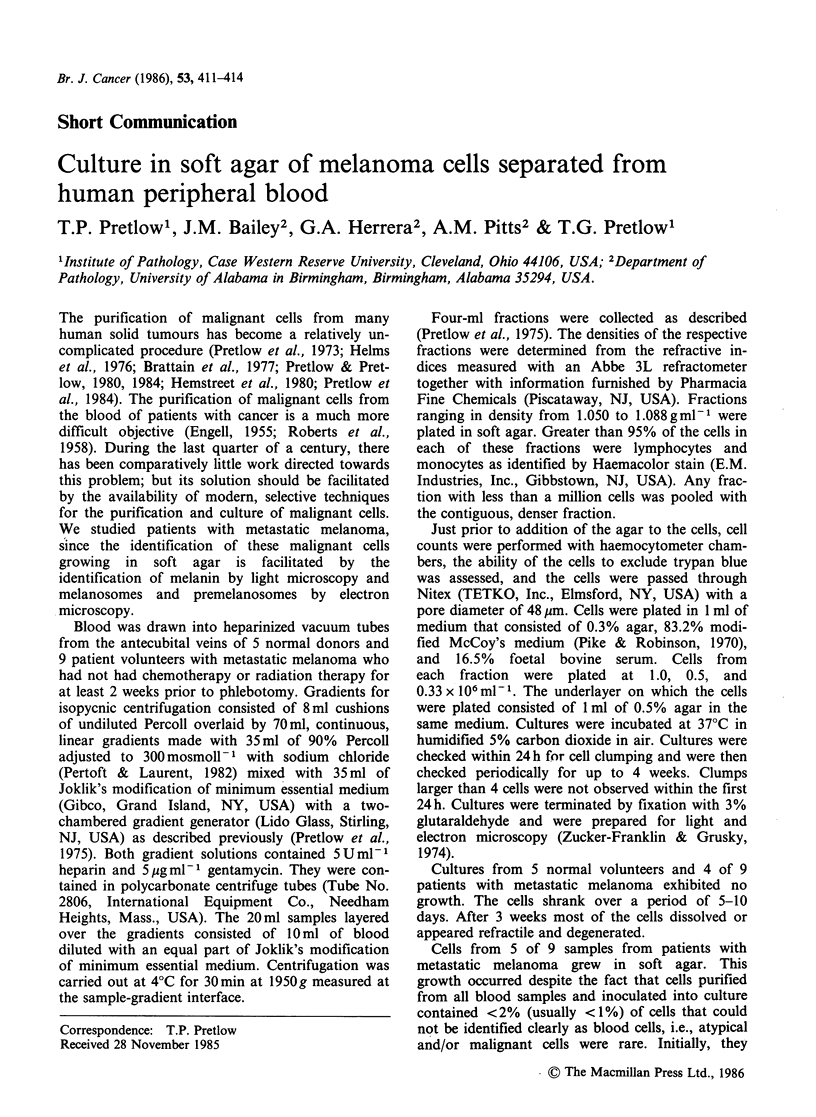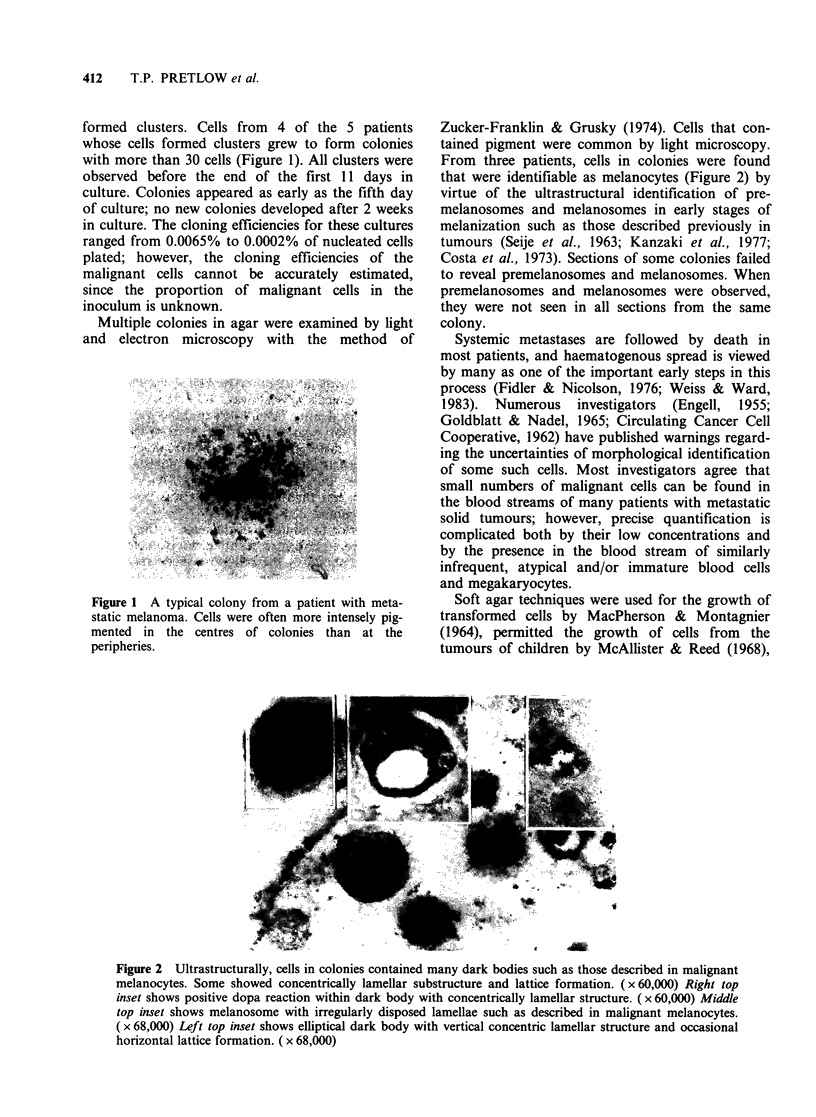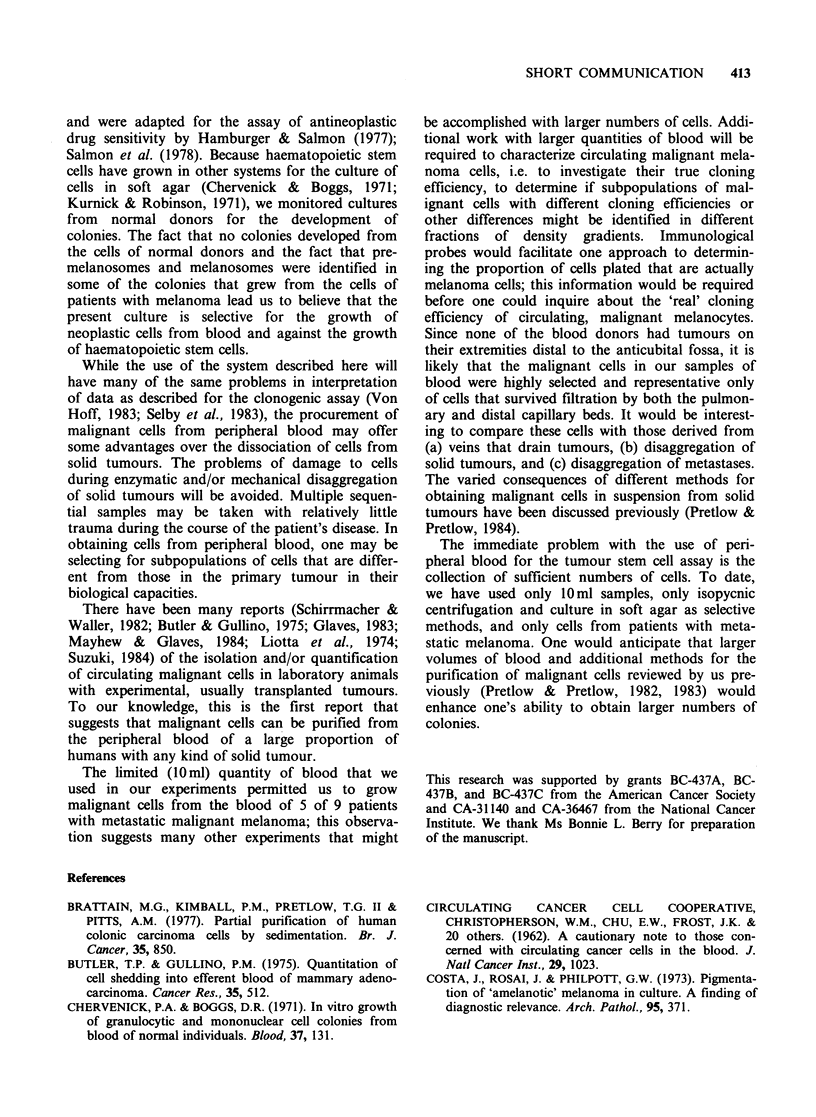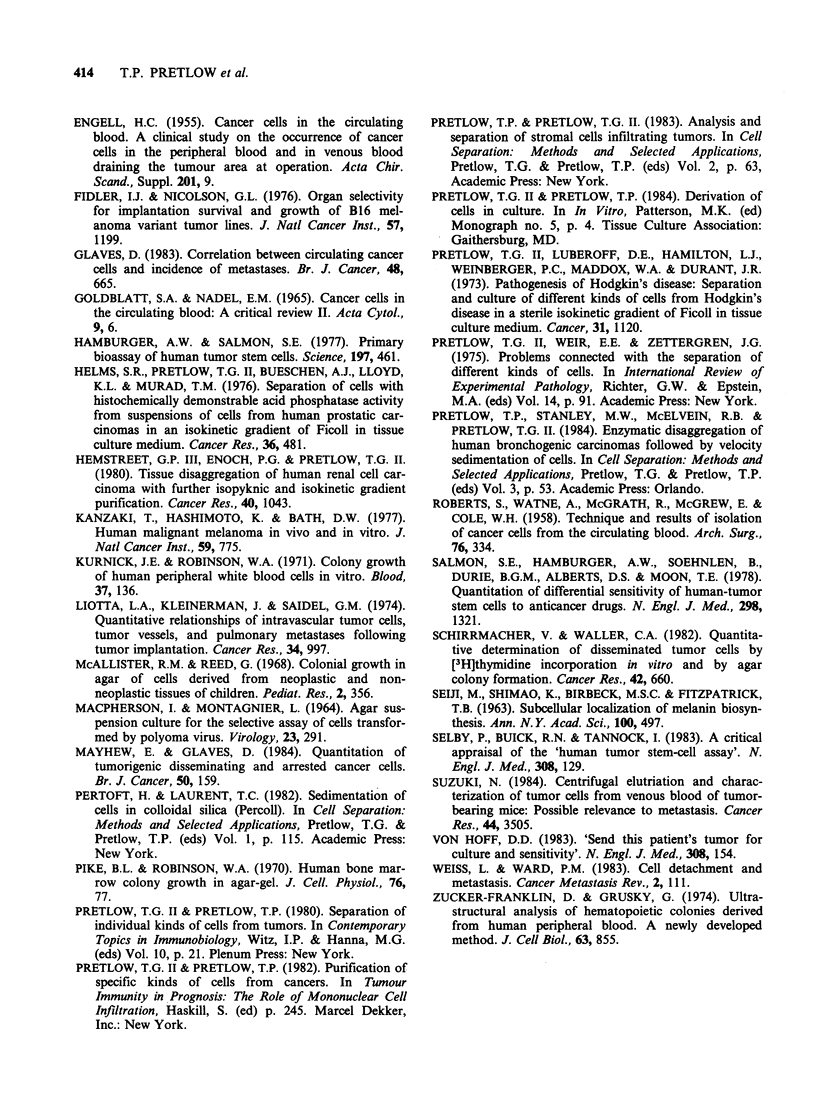# Culture in soft agar of melanoma cells separated from human peripheral blood.

**DOI:** 10.1038/bjc.1986.67

**Published:** 1986-03

**Authors:** T. P. Pretlow, J. M. Bailey, G. A. Herrera, A. M. Pitts, T. G. Pretlow

## Abstract

**Images:**


*Br. J. Cancer* (1986), **53**, 411–414

### Short Communication

### Culture in soft agar of melanoma cells separated from human peripheral blood

T.P. Pretlow[1], J.M. Bailey[2], G.A. Herrera[2], A.M. Pitts[2] & T.G. Pretlow[1]

[1]*Institute of Pathology, Case Western Reserve University, Cleveland, Ohio 44106, USA;* [2]*Department of Pathology, University of Alabama in Birmingham, Birmingham, Alabama 35294, USA.*

The purification of malignant cells from many human solid tumours has become a relatively uncomplicated procedure (Pretlow *et al.*, 1973; Helms *et al.*, 1976; Brattain *et al.*, 1977; Pretlow & Pretlow, 1980, 1984; Hemstreet *et al.*, 1980; Pretlow *et al.*, 1984). The purification of malignant cells from the blood of patients with cancer is a much more difficult objective (Engell, 1955; Roberts *et al.*, 1958). During the last quarter of a century, there has been comparatively little work directed towards this problem; but its solution should be facilitated by the availability of modern, selective techniques for the purification and culture of malignant cells. We studied patients with metastatic melanoma, since the identification of these malignant cells growing in soft agar is facilitated by the identification of melanin by light microscopy and melanosomes and premelanosomes by electron microscopy.

Blood was drawn into heparinized vacuum tubes from the antecubital veins of 5 normal donors and 9 patient volunteers with metastatic melanoma who had not had chemotherapy or radiation therapy for at least 2 weeks prior to phlebotomy. Gradients for isopycnic centrifugation consisted of 8 ml cushions of undiluted Percoll overlaid by 70 ml, continuous, linear gradients made with 35 ml of 90% Percoll adjusted to 300 mosmol $l^{-1}$ with sodium chloride (Pertoft & Laurent, 1982) mixed with 35 ml of Joklik's modification of minimum essential medium (Gibco, Grand Island, NY, USA) with a two-chambered gradient generator (Lido Glass, Stirling, NJ, USA) as described previously (Pretlow *et al.*, 1975). Both gradient solutions contained 5 U $ml^{-1}$ heparin and 5 $\mu g\ ml^{-1}$ gentamycin. They were contained in polycarbonate centrifuge tubes (Tube No. 2806, International Equipment Co., Needham Heights, Mass., USA). The 20 ml samples layered over the gradients consisted of 10 ml of blood diluted with an equal part of Joklik's modification of minimum essential medium. Centrifugation was carried out at 4°C for 30 min at 1950 *g* measured at the sample-gradient interface.

Correspondence: T.P. Pretlow
Received 28 November 1985

Four-ml fractions were collected as described (Pretlow *et al.*, 1975). The densities of the respective fractions were determined from the refractive indices measured with an Abbe 3L refractometer together with information furnished by Pharmacia Fine Chemicals (Piscataway, NJ, USA). Fractions ranging in density from 1.050 to 1.088 g $ml^{-1}$ were plated in soft agar. Greater than 95% of the cells in each of these fractions were lymphocytes and monocytes as identified by Haemacolor stain (E.M. Industries, Inc., Gibbstown, NJ, USA). Any fraction with less than a million cells was pooled with the contiguous, denser fraction.

Just prior to addition of the agar to the cells, cell counts were performed with haemocytometer chambers, the ability of the cells to exclude trypan blue was assessed, and the cells were passed through Nitex (TETKO, Inc., Elmsford, NY, USA) with a pore diameter of 48 $\mu$m. Cells were plated in 1 ml of medium that consisted of 0.3% agar, 83.2% modified McCoy's medium (Pike & Robinson, 1970), and 16.5% foetal bovine serum. Cells from each fraction were plated at 1.0, 0.5, and $0.33 \times 10^6\ ml^{-1}$. The underlayer on which the cells were plated consisted of 1 ml of 0.5% agar in the same medium. Cultures were incubated at 37°C in humidified 5% carbon dioxide in air. Cultures were checked within 24 h for cell clumping and were then checked periodically for up to 4 weeks. Clumps larger than 4 cells were not observed within the first 24 h. Cultures were terminated by fixation with 3% glutaraldehyde and were prepared for light and electron microscopy (Zucker-Franklin & Grusky, 1974).

Cultures from 5 normal volunteers and 4 of 9 patients with metastatic melanoma exhibited no growth. The cells shrank over a period of 5–10 days. After 3 weeks most of the cells dissolved or appeared refractile and degenerated.

Cells from 5 of 9 samples from patients with metastatic melanoma grew in soft agar. This growth occurred despite the fact that cells purified from all blood samples and inoculated into culture contained <2% (usually <1%) of cells that could not be identified clearly as blood cells, i.e., atypical and/or malignant cells were rare. Initially, they

© The Macmillan Press Ltd., 1986

formed clusters. Cells from 4 of the 5 patients whose cells formed clusters grew to form colonies with more than 30 cells (Figure 1). All clusters were observed before the end of the first 11 days in culture. Colonies appeared as early as the fifth day of culture; no new colonies developed after 2 weeks in culture. The cloning efficiencies for these cultures ranged from 0.0065% to 0.0002% of nucleated cells plated; however, the cloning efficiencies of the malignant cells cannot be accurately estimated, since the proportion of malignant cells in the inoculum is unknown.

Multiple colonies in agar were examined by light and electron microscopy with the method of Zucker-Franklin & Grusky (1974). Cells that contained pigment were common by light microscopy. From three patients, cells in colonies were found that were identifiable as melanocytes (Figure 2) by virtue of the ultrastructural identification of premelanosomes and melanosomes in early stages of melanization such as those described previously in tumours (Seije *et al.*, 1963; Kanzaki *et al.*, 1977; Costa *et al.*, 1973). Sections of some colonies failed to reveal premelanosomes and melanosomes. When premelanosomes and melanosomes were observed, they were not seen in all sections from the same colony.

**Figure 1** A typical colony from a patient with metastatic melanoma. Cells were often more intensely pigmented in the centres of colonies than at the peripheries.

Systemic metastases are followed by death in most patients, and haematogenous spread is viewed by many as one of the important early steps in this process (Fidler & Nicolson, 1976; Weiss & Ward, 1983). Numerous investigators (Engell, 1955; Goldblatt & Nadel, 1965; Circulating Cancer Cell Cooperative, 1962) have published warnings regarding the uncertainties of morphological identification of some such cells. Most investigators agree that small numbers of malignant cells can be found in the blood streams of many patients with metastatic solid tumours; however, precise quantification is complicated both by their low concentrations and by the presence in the blood stream of similarly infrequent, atypical and/or immature blood cells and megakaryocytes.

Soft agar techniques were used for the growth of transformed cells by MacPherson & Montagnier (1964), permitted the growth of cells from the tumours of children by McAllister & Reed (1968),

**Figure 2** Ultrastructurally, cells in colonies contained many dark bodies such as those described in malignant melanocytes. Some showed concentrically lamellar substructure and lattice formation. (×60,000) ***Right top inset*** shows positive dopa reaction within dark body with concentrically lamellar structure. (×60,000) ***Middle top inset*** shows melanosome with irregularly disposed lamellae such as described in malignant melanocytes. (×68,000) ***Left top inset*** shows elliptical dark body with vertical concentric lamellar structure and occasional horizontal lattice formation. (×68,000)

and were adapted for the assay of antineoplastic drug sensitivity by Hamburger & Salmon (1977); Salmon *et al.* (1978). Because haematopoietic stem cells have grown in other systems for the culture of cells in soft agar (Chervenick & Boggs, 1971; Kurnick & Robinson, 1971), we monitored cultures from normal donors for the development of colonies. The fact that no colonies developed from the cells of normal donors and the fact that premelanosomes and melanosomes were identified in some of the colonies that grew from the cells of patients with melanoma lead us to believe that the present culture is selective for the growth of neoplastic cells from blood and against the growth of haematopoietic stem cells.

While the use of the system described here will have many of the same problems in interpretation of data as described for the clonogenic assay (Von Hoff, 1983; Selby *et al.*, 1983), the procurement of malignant cells from peripheral blood may offer some advantages over the dissociation of cells from solid tumours. The problems of damage to cells during enzymatic and/or mechanical disaggregation of solid tumours will be avoided. Multiple sequential samples may be taken with relatively little trauma during the course of the patient's disease. In obtaining cells from peripheral blood, one may be selecting for subpopulations of cells that are different from those in the primary tumour in their biological capacities.

There have been many reports (Schirrmacher & Waller, 1982; Butler & Gullino, 1975; Glaves, 1983; Mayhew & Glaves, 1984; Liotta *et al.*, 1974; Suzuki, 1984) of the isolation and/or quantification of circulating malignant cells in laboratory animals with experimental, usually transplanted tumours. To our knowledge, this is the first report that suggests that malignant cells can be purified from the peripheral blood of a large proportion of humans with any kind of solid tumour.

The limited (10 ml) quantity of blood that we used in our experiments permitted us to grow malignant cells from the blood of 5 of 9 patients with metastatic malignant melanoma; this observation suggests many other experiments that might be accomplished with larger numbers of cells. Additional work with larger quantities of blood will be required to characterize circulating malignant melanoma cells, i.e. to investigate their true cloning efficiency, to determine if subpopulations of malignant cells with different cloning efficiencies or other differences might be identified in different fractions of density gradients. Immunological probes would facilitate one approach to determining the proportion of cells plated that are actually melanoma cells; this information would be required before one could inquire about the 'real' cloning efficiency of circulating, malignant melanocytes. Since none of the blood donors had tumours on their extremities distal to the anticubital fossa, it is likely that the malignant cells in our samples of blood were highly selected and representative only of cells that survived filtration by both the pulmonary and distal capillary beds. It would be interesting to compare these cells with those derived from (a) veins that drain tumours, (b) disaggregation of solid tumours, and (c) disaggregation of metastases. The varied consequences of different methods for obtaining malignant cells in suspension from solid tumours have been discussed previously (Pretlow & Pretlow, 1984).

The immediate problem with the use of peripheral blood for the tumour stem cell assay is the collection of sufficient numbers of cells. To date, we have used only 10 ml samples, only isopycnic centrifugation and culture in soft agar as selective methods, and only cells from patients with metastatic melanoma. One would anticipate that larger volumes of blood and additional methods for the purification of malignant cells reviewed by us previously (Pretlow & Pretlow, 1982, 1983) would enhance one's ability to obtain larger numbers of colonies.

This research was supported by grants BC-437A, BC-437B, and BC-437C from the American Cancer Society and CA-31140 and CA-36467 from the National Cancer Institute. We thank Ms Bonnie L. Berry for preparation of the manuscript.

**References**

BRATTAIN, M.G., KIMBALL, P.M., PRETLOW, T.G. II & PITTS, A.M. (1977). Partial purification of human colonic carcinoma cells by sedimentation. *Br. J. Cancer*, **35**, 850.

BUTLER, T.P. & GULLINO, P.M. (1975). Quantitation of cell shedding into efferent blood of mammary adenocarcinoma. *Cancer Res.*, **35**, 512.

CHERVENICK, P.A. & BOGGS, D.R. (1971). In vitro growth of granulocytic and mononuclear cell colonies from blood of normal individuals. *Blood*, **37**, 131.

CIRCULATING CANCER CELL COOPERATIVE, CHRISTOPHERSON, W.M., CHU, E.W., FROST, J.K. & 20 others. (1962). A cautionary note to those concerned with circulating cancer cells in the blood. *J. Natl Cancer Inst.*, **29**, 1023.

COSTA, J., ROSAI, J. & PHILPOTT, G.W. (1973). Pigmentation of 'amelanotic' melanoma in culture. A finding of diagnostic relevance. *Arch. Pathol.*, **95**, 371.

ENGELL, H.C. (1955). Cancer cells in the circulating blood. A clinical study on the occurrence of cancer cells in the peripheral blood and in venous blood draining the tumour area at operation. *Acta Chir. Scand.*, Suppl. **201**, 9.

FIDLER, I.J. & NICOLSON, G.L. (1976). Organ selectivity for implantation survival and growth of B16 melanoma variant tumor lines. *J. Natl Cancer Inst.*, **57**, 1199.

GLAVES, D. (1983). Correlation between circulating cancer cells and incidence of metastases. *Br. J. Cancer*, **48**, 665.

GOLDBLATT, S.A. & NADEL, E.M. (1965). Cancer cells in the circulating blood: A critical review II. *Acta Cytol.*, **9**, 6.

HAMBURGER, A.W. & SALMON, S.E. (1977). Primary bioassay of human tumor stem cells. *Science*, **197**, 461.

HELMS, S.R., PRETLOW, T.G. II, BUESCHEN, A.J., LLOYD, K.L. & MURAD, T.M. (1976). Separation of cells with histochemically demonstrable acid phosphatase activity from suspensions of cells from human prostatic carcinomas in an isokinetic gradient of Ficoll in tissue culture medium. *Cancer Res.*, **36**, 481.

HEMSTREET, G.P. III, ENOCH, P.G. & PRETLOW, T.G. II. (1980). Tissue disaggregation of human renal cell carcinoma with further isopyknic and isokinetic gradient purification. *Cancer Res.*, **40**, 1043.

KANZAKI, T., HASHIMOTO, K. & BATH, D.W. (1977). Human malignant melanoma in vivo and in vitro. *J. Natl Cancer Inst.*, **59**, 775.

KURNICK, J.E. & ROBINSON, W.A. (1971). Colony growth of human peripheral white blood cells in vitro. *Blood*, **37**, 136.

LIOTTA, L.A., KLEINERMAN, J. & SAIDEL, G.M. (1974). Quantitative relationships of intravascular tumor cells, tumor vessels, and pulmonary metastases following tumor implantation. *Cancer Res.*, **34**, 997.

McALLISTER, R.M. & REED, G. (1968). Colonial growth in agar of cells derived from neoplastic and non-neoplastic tissues of children. *Pediat. Res.*, **2**, 356.

MACPHERSON, I. & MONTAGNIER, L. (1964). Agar suspension culture for the selective assay of cells transformed by polyoma virus. *Virology*, **23**, 291.

MAYHEW, E. & GLAVES, D. (1984). Quantitation of tumorigenic disseminating and arrested cancer cells. *Br. J. Cancer*, **50**, 159.

PERTOFT, H. & LAURENT, T.C. (1982). Sedimentation of cells in colloidal silica (Percoll). In *Cell Separation: Methods and Selected Applications*, Pretlow, T.G. & Pretlow, T.P. (eds) Vol. 1, p. 115. Academic Press: New York.

PIKE, B.L. & ROBINSON, W.A. (1970). Human bone marrow colony growth in agar-gel. *J. Cell. Physiol.*, **76**, 77.

PRETLOW, T.G. II & PRETLOW, T.P. (1980). Separation of individual kinds of cells from tumors. In *Contemporary Topics in Immunobiology*, Witz, I.P. & Hanna, M.G. (eds) Vol. 10, p. 21. Plenum Press: New York.

PRETLOW, T.G. II & PRETLOW, T.P. (1982). Purification of specific kinds of cells from cancers. In *Tumour Immunity in Prognosis: The Role of Mononuclear Cell Infiltration*, Haskill, S. (ed) p. 245. Marcel Dekker, Inc.: New York.

PRETLOW, T.P. & PRETLOW, T.G. II. (1983). Analysis and separation of stromal cells infiltrating tumors. In *Cell Separation: Methods and Selected Applications*, Pretlow, T.G. & Pretlow, T.P. (eds) Vol. 2, p. 63, Academic Press: New York.

PRETLOW, T.G. II & PRETLOW, T.P. (1984). Derivation of cells in culture. In *In Vitro*, Patterson, M.K. (ed) Monograph no. 5, p. 4. Tissue Culture Association: Gaithersburg, MD.

PRETLOW, T.G. II, LUBEROFF, D.E., HAMILTON, L.J., WEINBERGER, P.C., MADDOX, W.A. & DURANT, J.R. (1973). Pathogenesis of Hodgkin's disease: Separation and culture of different kinds of cells from Hodgkin's disease in a sterile isokinetic gradient of Ficoll in tissue culture medium. *Cancer*, **31**, 1120.

PRETLOW, T.G. II, WEIR, E.E. & ZETTERGREN, J.G. (1975). Problems connected with the separation of different kinds of cells. In *International Review of Experimental Pathology*, Richter, G.W. & Epstein, M.A. (eds) Vol. 14, p. 91. Academic Press: New York.

PRETLOW, T.P., STANLEY, M.W., McELVEIN, R.B. & PRETLOW, T.G. II. (1984). Enzymatic disaggregation of human bronchogenic carcinomas followed by velocity sedimentation of cells. In *Cell Separation: Methods and Selected Applications*, Pretlow, T.G. & Pretlow, T.P. (eds) Vol. 3, p. 53. Academic Press: Orlando.

ROBERTS, S., WATNE, A., McGRATH, R., McGREW, E. & COLE, W.H. (1958). Technique and results of isolation of cancer cells from the circulating blood. *Arch. Surg.*, **76**, 334.

SALMON, S.E., HAMBURGER, A.W., SOEHNLEN, B., DURIE, B.G.M., ALBERTS, D.S. & MOON, T.E. (1978). Quantitation of differential sensitivity of human-tumor stem cells to anticancer drugs. *N. Engl. J. Med.*, **298**, 1321.

SCHIRRMACHER, V. & WALLER, C.A. (1982). Quantitative determination of disseminated tumor cells by [$^3$H]thymidine incorporation *in vitro* and by agar colony formation. *Cancer Res.*, **42**, 660.

SEIJI, M., SHIMAO, K., BIRBECK, M.S.C. & FITZPATRICK, T.B. (1963). Subcellular localization of melanin biosynthesis. *Ann. N.Y. Acad. Sci.*, **100**, 497.

SELBY, P., BUICK, R.N. & TANNOCK, I. (1983). A critical appraisal of the 'human tumor stem-cell assay'. *N. Engl. J. Med.*, **308**, 129.

SUZUKI, N. (1984). Centrifugal elutriation and characterization of tumor cells from venous blood of tumor-bearing mice: Possible relevance to metastasis. *Cancer Res.*, **44**, 3505.

VON HOFF, D.D. (1983). 'Send this patient's tumor for culture and sensitivity'. *N. Engl. J. Med.*, **308**, 154.

WEISS, L. & WARD, P.M. (1983). Cell detachment and metastasis. *Cancer Metastasis Rev.*, **2**, 111.

ZUCKER-FRANKLIN, D. & GRUSKY, G. (1974). Ultrastructural analysis of hematopoietic colonies derived from human peripheral blood. A newly developed method. *J. Cell Biol.*, **63**, 855.